# UBC-Nepal Expedition: An experimental overview of the 2016 University of British Columbia Scientific Expedition to Nepal Himalaya

**DOI:** 10.1371/journal.pone.0204660

**Published:** 2018-10-31

**Authors:** Christopher K. Willie, Michael Stembridge, Ryan L. Hoiland, Michael M. Tymko, Joshua C. Tremblay, Alexander Patrician, Craig Steinback, Jonathan Moore, James Anholm, Prajan Subedi, Shailesh Niroula, Chris J. McNeil, Ali McManus, David B. MacLeod, Philip N. Ainslie

**Affiliations:** 1 Centre for Heart, Lung and Vascular Health, School of Health and Exercise Sciences, University of British Columbia – Okanagan, Kelowna, British Columbia, Canada; 2 Cardiff Centre for Exercise and Health, Cardiff School of Sport, Cardiff Metropolitan University, Cardiff, United Kingdom; 3 Cardiovascular Stress Response Laboratory, School of Kinesiology and Health Studies, Queen’s University, Kingston, Ontario, Canada; 4 University of Alberta, Edmonton, Canada; 5 Bangor University, School of Sport, Health & Exercise Sciences, Gwynedd, Wales, United Kingdom; 6 Pulmonary/Critical Care Section, VA Loma Linda Healthcare System, Loma Linda, California, United States of America; 7 Paloma Medical Group, San Juan Capistrano, California, United States of America; 8 Institute of Medicine, Tribhuvan University, Kathmandu, Nepal; 9 Human Pharmacology & Physiology Lab, Duke University Medical Center, Durham, North Carolina, United States of America; University of Southampton, UNITED KINGDOM

## Abstract

The University of British Columbia Nepal Expedition took place over several months in the fall of 2016 and was comprised of an international team of 37 researchers. This paper describes the objectives, study characteristics, organization and management of this expedition, and presents novel blood gas data during acclimatization in both lowlanders and Sherpa. An overview and framework for the forthcoming publications is provided. The expedition conducted 17 major studies with two principal goals—to identify physiological differences in: 1) acclimatization; and 2) responses to sustained high-altitude exposure between lowland natives and people of Tibetan descent. We performed observational cohort studies of human responses to progressive hypobaric hypoxia (during ascent), and to sustained exposure to 5050 m over 3 weeks comparing lowlander adults (n = 30) with Sherpa adults (n = 24). Sherpa were tested both with (n = 12) and without (n = 12) descent to Kathmandu. Data collected from lowlander children (n = 30) in Canada were compared with those collected from Sherpa children (n = 57; 3400–3900m). Studies were conducted in Canada (344m) and the following locations in Nepal: Kathmandu (1400m), Namche Bazaar (3440m), Kunde Hospital (3480m), Pheriche (4371m) and the Ev-K2-CNR Research Pyramid Laboratory (5050m). The core studies focused on the mechanisms of cerebral blood flow regulation, the role of iron in cardiopulmonary regulation, pulmonary pressures, intra-ocular pressures, cardiac function, neuromuscular fatigue and function, blood volume regulation, autonomic control, and micro and macro vascular function. A total of 335 study sessions were conducted over three weeks at 5050m. In addition to an overview of this expedition and arterial blood gas data from Sherpa, suggestions for scientists aiming to perform field-based altitude research are also presented. Together, these findings will contribute to our understanding of human acclimatization and adaptation to the stress of residence at high-altitude.

## Introduction

The study of human physiology during acute and chronic exposure to high-altitude informs our understanding of the physical response to reduced oxygen availability. Hypoxemia, such as that experienced at high-altitude, is also a stress common amongst critically and chronically ill patients. There is, however, a markedly heterogeneous response between individuals to the same stimuli [1,2]. At sea-level, simulated hypoxic stress can be achieved through exposure to a reduced inspired oxygen fraction in an enclosed environment (e.g. hypoxic chambers or tents) and is a useful laboratory based approach for the study of acute hypoxic exposure. Such an approach is problematic for the study of acclimatization processes and long-term hypoxic exposure. This is predominately because of the complexities of conducting a lengthy study in the confines of a small chamber. Field expeditions have therefore long been the *modus operandi* of physiologists interested in the physiological responses to high-altitude [3]. These expeditions are notable, not just for their substantial contributions to the natural sciences, but also for the logistical hurdles and physical hardship of living and conducting research at altitude.

Understanding biological adaptation to the chronic environmental stress of living at high-altitude necessitates a long period of study and, ideally, comparison between sea-level natives and humans native to high-altitude. In high-altitude populations, modernization, migration, and the consequent genetic admixture is rapidly occurring; therefore, study of the physiological ramifications of human evolution to high-altitude is of immediate scientific importance [4]. Approximately 40,000 years ago, a human migration into the Tibetan plateau occurred with high incidence of two gene variants acquired through admixture with Denisovan populations [5]. This distinct genetic haplotype facilitates higher birth weight, lower infant mortality, reduced hematocrit, and the legendary exercise performance at high-altitude for which the Sherpa people of the Khumbu region of Nepal are famous. However, delineation of the specific mechanisms involved in their superior physiological function at altitude remains undefined, and the window of opportunity to study these mechanisms is quickly closing.

Of the multitude of physiological adaptations that occur upon ascent to altitude, increased alveolar ventilation is the most important. This response mitigates the drop in the partial pressure of arterial oxygen (PaO_2_) by increasing alveolar PO_2_ at any given inspired PO_2_. Despite the importance of this adaptation, the phenotypic differences related to alveolar ventilation at altitude that may (or may not) be present between Sherpa and lowlanders have yet to be clearly demonstrated, with variable findings throughout the literature (reviewed in: [6]).

The purpose of this manuscript is two-fold: First, it aims to provide a summary of the 2016 University of British Columbia (UBC) expedition to the Khumbu region of the Nepal Himalaya. The principal scientific themes of the expedition along with the more notable aspects of its execution are discussed herein. Given the logistical nature of high-altitude expeditions, data overlap for independent variables is often unavoidable due to the costs associated with repeated measurement/sampling (e.g., extra consumables, shipping, disposal) [7]. Thus, for the purposes of transparency we present the arterial blood gas data upon ascent to altitude for all subjects measured, including the Sherpa; the results and specific primary outcome variables of individual and experimentally unique studies will be published as discrete papers. Second, we aim to highlight phenotypic differences in arterial blood gases between Sherpa and lowlanders during ascent to altitude to provide a novel insight into the effect of race on the acclimatization process to high-altitude.

## Methods

### Overview

The UBC Nepal Expedition was undertaken in September to November 2016, with baseline studies conducted over the two months prior. The research group was comprised of 37 researchers with key leadership from UBC, Cardiff Metropolitan University, University of Alberta, Duke University Medical Centre, Loma Linda University, University of Cambridge, and Bangor University. Studies were designed to either measure a physiological change from (i) a baseline elevation (344m, UBC, Kelowna, BC, Canada in lowlanders; 1400m, Kathmandu, in Sherpa), (ii) a difference between Sherpa and lowlanders during ascent or after acclimatization at 5050m, or (iii) a difference before and after a pharmacological intervention(s) at altitude (5050m, Ev-K2-CNR Research Pyramid Laboratory, Khumbu Valley, Nepal). In total, 335 study sessions comprising 17 distinct studies were completed over three weeks, an overview of which is given in the text and Table 1.

**Table 1:** Core studies conducted on the UBC expedition, including objectives, key measures and sample sizes.

Study	Study Title	Aim	Sample size	Intervention / techniques
***Ascent studies***				
1	Comparative effects on the pulmonary vasculature of ascent to high-altitude in lowlanders and high-altitude natives.	To characterize pulmonary arterial and right ventricular function during ascent to high-altitude, and how these parameters are ameliorated by supplemental oxygen.	19 lowlander 12 Sherpa	Echocardiography; O_2_ supplement; blood pressure.
2	Cerebral vascular regulation in lowlanders and Sherpa upon ascent to 5050m.	To assess if evolutionary adaptation to hypoxia is reflected in phenotypical differences in CBF regulation between lowlander and Sherpa during graded hypoxia.	21 lowlanders 23 Sherpa	Duplex ultrasound; blood pressure; blood gases.
3	A non-invasive approach to the pathophysiology of acute mountain sickness.	To assess the predictive relationship between optic nerve sheath diameter and acute mountain sickness using known physiological ramifications of ascent to high-altitude.	30 lowlanders	ONSD; head-down tilt; blood pressure.
4	Peripheral vascular function in lowlanders and Sherpa upon ascent to 5050m.	To assess if evolutionary adaptation to hypoxia is reflected in phenotypical differences in peripheral vascular regulation between lowlander and Sherpa during graded hypoxia.	22 lowlander 12 Sherpa	Duplex ultrasound; blood pressure; blood gases.
5	Iron metabolism during ascent to high-altitude: lowlanders versus high-altitude natives.	To examine changes in iron regulation during ascent to 5050m in lowland and highland natives.	21 lowlanders 12 Sherpa	Echocardiography; O_2_ supplement; blood pressure; arterial and venous blood samples.
6	Cerebral autoregulation during transient hypotension.	To examine the cerebral blood flow response during a brief reduction in blood pressure in both lowlanders and high-altitude natives upon ascent to 5050m.	10 lowlanders 10 Sherpa	Duplex ultrasound; O_2_ supplement; blood pressure.
***Laboratory studies***				
7	Central effects of exercise in Sherpa children at high-altitude.	To determine resting regional and global cerebral blood flow in Sherpa children living at high-altitude and lowlander children residing at sea-level and ii) to characterize the effects of progressive exercise to exhaustion on ventilation and cerebral blood flow velocity in Sherpa children at high-altitude and lowlander children residing at sea-level.	30 lowland children 57 Sherpa Children	Duplex ultrasound; blood pressure; respiratory gas exchange; cycle ergometer.
8	Neuromuscular fatigue in lowlanders and Sherpa upon ascent to 5050m.	To assess the impact of hypoxia and acclimatization on fatigue-induced changes within the central nervous system and the muscle in lowlanders and Sherpa.	12 lowlanders 10 Sherpa	Surface EMG; isometric myograph; muscle-belly stimulation; TMS; CMS; BPS; duplex ultrasound; NIRS.
9	Motor control and adaptation to high-altitude.	To assess motor unit behaviour and motor performance in lowlanders and Sherpa.	11 lowlanders 11 Sherpa	Intramuscular and surface EMG, isometric myograph.
10	The role of iron and the hypoxia-inducible factor system in the pulmonary vascular response to altitude.	To examine the role of iron in raised pulmonary arterial pressures in hypoxia and to compare between Sherpa and lowlanders.	20 lowlander 19 Sherpa	Echocardiography; O_2_ supplement; hypoxic gas; blood pressure; arterial and venous blood samples; cycle ergometer; *iv*–Iron (200mg) or DFO (4g).
11	Sympathetic function at high-altitude: lowlanders versus high-altitude natives.	To examine the effect of acute and chronic hypoxia on sympathetic activity and neural transduction and to contrast the impact of hypoxia on lowlanders and high-altitude natives.	14 lowlander 8 Sherpa	Vascular ultrasound; O_2_ supplement; hypoxic gas mix; blood pressure; arterial and venous blood samples; microneurography. Oxford technique [SNP (20ug / L blood volume) vs PE (30ug / L blood volume)].
12	Oxidative stress and cerebral blood flow at high-altitude.	To examine the role of oxidative stress on cerebrovascular function during acute and chronic hypoxia in humans.	16 lowlanders	Duplex ultrasound; TCD; blood pressure; respiration; oral antioxidants (500 mg vitamin C, 400 IU vitamin E and 300mg -αlipoic acid).
13	The mechanisms governing oxygen content mediated regulation of cerebral blood flow during acute and chronic hypoxia.	To determine the role of arterial oxygen content versus arterial oxygen tension in regulating cerebral blood flow in acute and chronic hypoxia.	17 lowlanders	Vascular ultrasound; O_2_ supplement; hypoxic gas; blood pressure; arterial and venous blood samples; hemodilution.
14	Shear stress and the endothelium during acute and chronic hypoxia in humans.	To determine whether endothelial function is preserved or worsened by periods of imposed retrograde shear stress during acute and chronic hypoxia.	15 lowlanders	Vascular ultrasound; venous blood sample.
15	The role of absolute blood volume and cardiac function in limiting maximal exercise performance in Sherpa.	To assess absolute blood volume in high-altitude Sherpa, and investigate the relationships between blood volume, hemoglobin mass and cardiac structure and function with maximal exercise capacity.	12 Sherpa	Echocardiography; blood pressure; arterial and venous blood samples; cycle ergometer. Blood volume was assessed at sea-level using the carbon monoxide rebreathing method [8], as previously used at high-altitude [9] (1ml kg^-1^ of CO).
16	The role of ß-adrenergic-dependent and–independent factors in the regulation of left ventricular twist in hypoxia.	To investigate the independent and combined influences of altered O_2_ saturation and adrenergic stimulation on left ventricular twist mechanics in hypoxic environments.	20 lowlanders	Echocardiography; blood pressure; infusion of Esmolol (cardiac specific β_1_-adrenergic receptor antagonist) as a 500 μg/kg bolus over 1 minute followed by 150 μg/kg/min continuous maintenance infusion.
17	The role of sympathetic nervous activity on brachial artery endothelial function at sea-level and high-altitude.	To determine the effects of acute and mild alterations in sympathetic nervous activity via lower-body differential pressure on vascular function assessed via brachial artery flow-mediated dilation.	15 lowlanders	Vascular ultrasound; blood pressure; LBNP/PP box.

Abbreviations: BPS, brachial plexus stimulation; cervicomedullary stimulation; CO, carbon monoxide; DFO, desferrioxamine; EMG, electromyography; LBNP/PP, lower body negative pressure / positive pressure; NIRS; near infrared spectroscopy; ONSD, optic nerve sheath diameter; PE, phenylephrine; SNP, sodium nitroprusside; TCD, transcranial doppler; TMS, transcranial magnetic stimulation.

### Subjects

**Fig 1:**
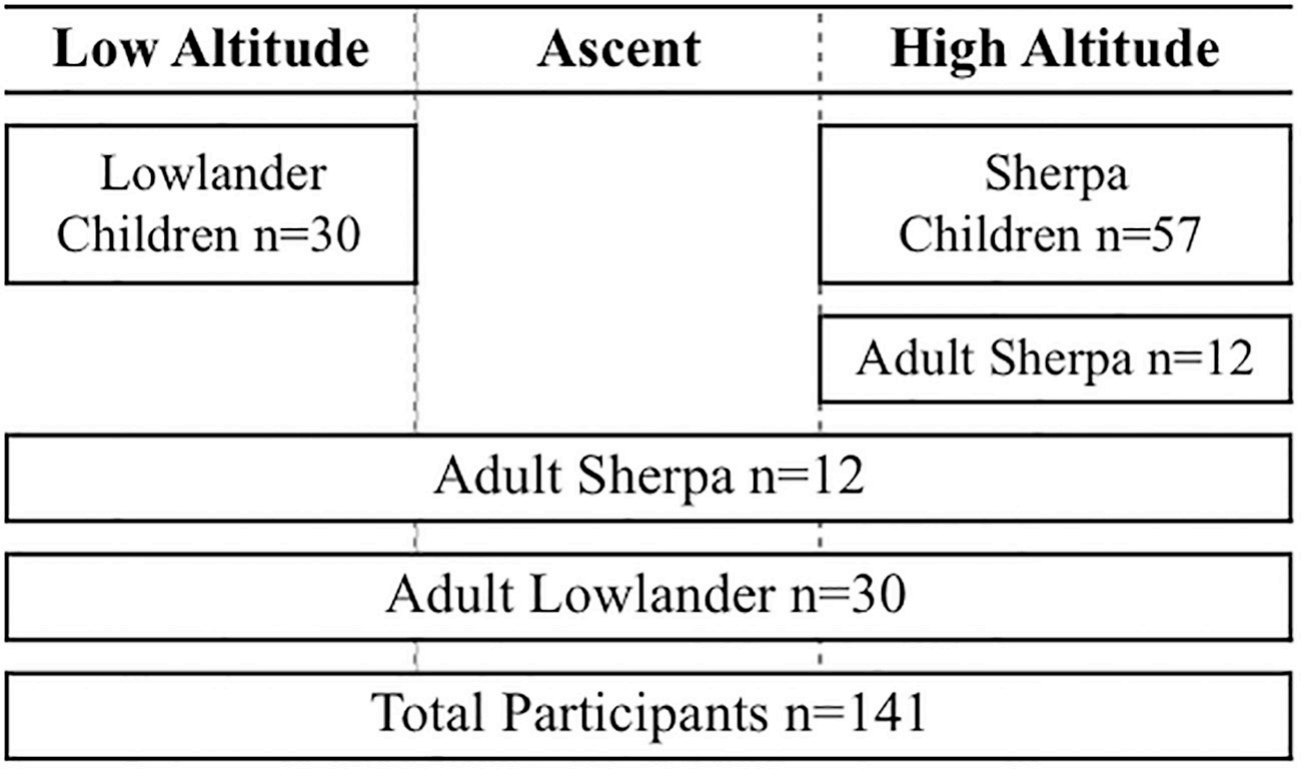
Schematic of sample sizes and location of study participation.

Fig 1 provides a schematic breakdown of the study cohort. The research participants for these studies were comprised of five groups totaling 141 participants (Fig 1): 1) 30 lowlander adults; 2) 12 Sherpa adults who de-acclimatized at 1400m for 9 ± 3 days (Age: 34±11, BMI: 24±4)—three of the Sherpa in this group summited Mount Everest (8848 m) in the previous year; 3) 57 Sherpa children; 4) 30 age and BMI-matched lowlander children tested in Canada at 344m; and, 5) 12 Sherpa adults (Age: 23±7, BMI: 21±2) who had not recently been below 3500m - these Sherpa reached a maximum altitude of 4800 m to 7800 m (median: 5545 m) in the last year.

All lowlanders were of European ancestry born at and living below 1000m, except one lowland Nepali born at 1400m but living at sea-level and without any known ancestry from native high-altitude populations. Studies had different sample sizes pooled from the same cohort. Lowland expedition members were included in 1–6 experiments, and thus visited the UBC laboratory on 2–6 occasions for the baseline arm of each study. Sherpa did not visit UBC; Sherpa baseline measures were collected either in Kathmandu (n = 12) for those individuals who were ascending to 5050m with the research team, or at 5050m preceding any interventional study (n = 12; see Table 1 and Fig 1 for individual study breakdown). Those 12 Sherpa that were part of the ascent studies arrived in Kathmandu 9 ± 3 days prior to re-ascent to altitude. Direct descendants of at least two known generations of Sherpa were recruited from local villages of the Solokhumbu valley by word of mouth. A detailed altitude history was collected from all Sherpa participants including altitudes *in utero*, at birth, during childhood, in adulthood, and for the 12 months preceding the studies. Prior to any study at low or high-altitude, participants abstained from exercise and caffeine for a minimum of 12 hours, and were fasted for at least two-hours. Due to the number of studies needing to be completed in a small period of time, 2–5 laboratories often ran simultaneously. Each study that involved a pharmacological intervention was separated from subsequent studies by at least five times the drug half-life to minimize any confounding influence of drugs across studies.

### Medical screening & safety

Thirty healthy lowlander adults were recruited from the members of the expedition (Table 2). Participants between the ages of 18–55 years without medical history of cardiopulmonary, cerebrovascular, or metabolic disease were considered for inclusion. Venous blood samples were collected and analyzed for complete blood count, serum iron, ferritin, and transferrin saturation. A Nepali physician (P. Subedi or S. Niroula) conducted complete medical histories for all Sherpa volunteers.

**Table 2:** Participant demographics and morphometrics.

	Lowland adults	Sherpa adults	P-value	Lowland children	Sherpa children	P-value
N (male/female)	25/5	24/0		16/14	28/29	
Age (years)	31±9	29±11	0.51	10±1	11±3	0.381
Height (cm)	176±8	169±6	<0.01	143±7	138±16	0.168
Weight (Kg)	73±10	64±11	<0.01	34±6	33±12	0.709
BMI (Kg/m^2^)	24±3	22±3	0.19	17±2	17±4	0.727

Mean ± StD.

### Ethical approval & consent

In accordance with the Declaration of Helsinki, the study was approved by both the UBC Clinical Research Ethics committee and Nepal Health Research Council (NHRC). This research was carried out within the framework of the Ev-K2-CNR laboratory in collaboration with the Nepal Academy of Science and Technology as foreseen in the Memorandum of Understanding between Nepal and Italy, with special thanks to a contribution from the Italian National Research Council. All potential participants signed the approved consent form—and for each child, consent was obtained from their respective parent or guardian. Prior to voluntary consent, opportunities for questions were offered at multiple stages in both countries. Sherpa adults and children were recruited through word of mouth and advertisement. An official Nepali translation of the consent form was provided with a Nepali physician present to explain and answer all questions. Further information was provided by one of three local Nepali clinical collaborators. In all locations throughout the expedition, the Nepali translators were present to allow for communication between Sherpa and investigators. All participants were free to withdraw without justification or penalty from all experiments at any time.

### Final preparations & ascent profile (Fig 2)

**Fig 2:**
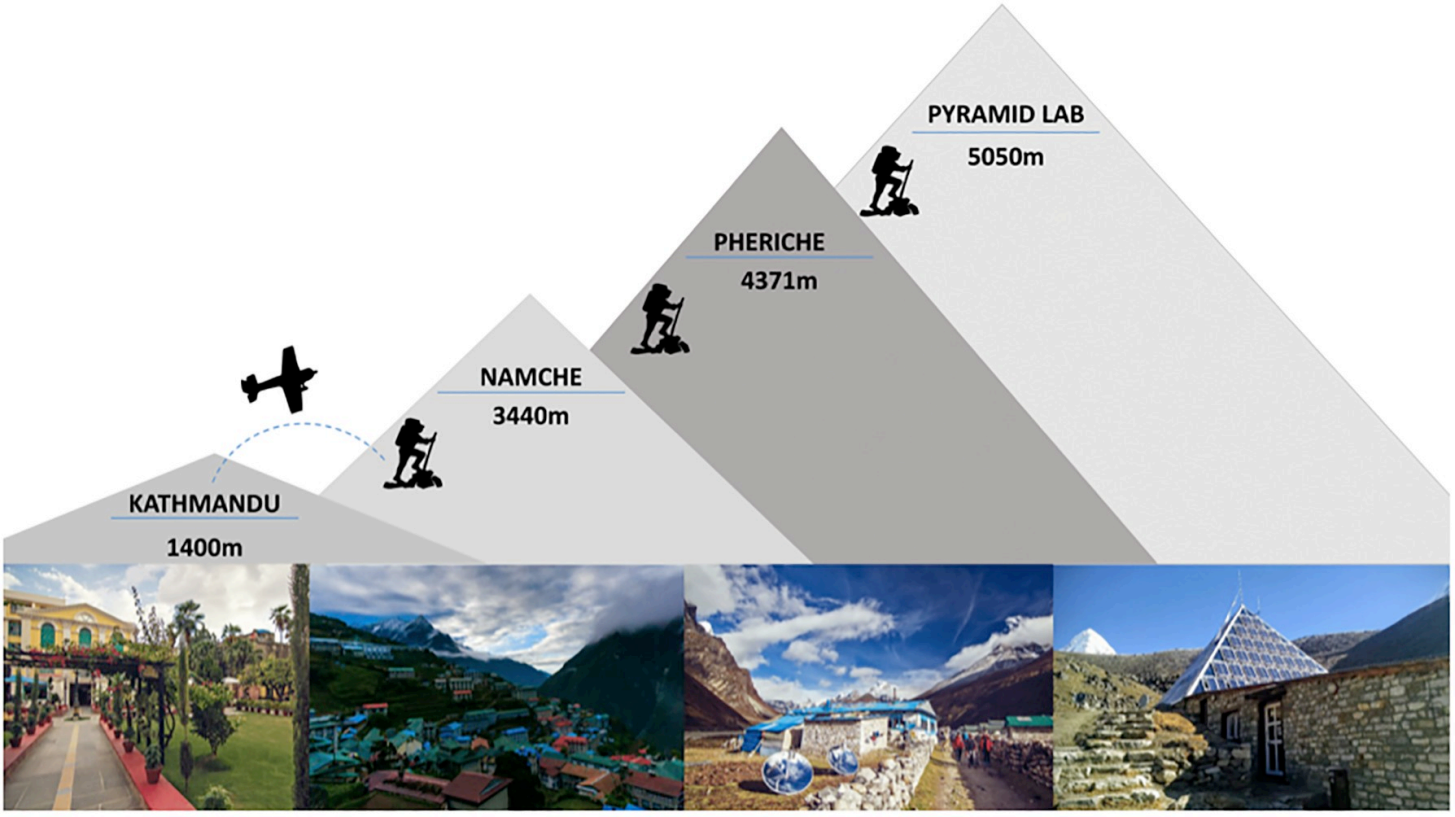
Ascent elevation profile for the UBC Scientific Expedition.

The expedition team assembled in Kathmandu (1400m) 3–9 days prior to departure to Lukla (2860m). This period was primarily devoted to Sherpa participant baseline testing and making final equipment preparations. For example, new uninterruptable power supply (UPS) devices had to be located when two UPS units caught fire in the hotel during baseline testing. While the availability of electricity in Kathmandu is now much better than ever before, future expeditions conducting testing are advised to find recently built/renovated hotels where wiring is more robust. The expedition medical kit, ~50L of saline, 100L of liquid nitrogen, and a miscellany of items from diesel to chocolate were purchased and added to the ~six tons of equipment to be flown and carried to 5050m. Transportation of liquid nitrogen was particularly problematic because: 1) commercial flights cannot technically provide transport, 2) it is difficult to convince local porters that a 100L metal drum releasing gas from the top is not, in fact, a bomb; and, 3) helicopters cannot technically carry such hazardous materials and are limited in the Khumbu to visual flight rules. Our shrewd expedition Sirdar was nonetheless able to facilitate the separate transport of two dewers of liquid nitrogen to and from the Pyramid Laboratory through a variety of means.

Expedition members flew to Lukla (2860m) over two days, after which they hiked as a group to the Pyramid Laboratory (5050m) over 9–10 days with obligatory rest / testing days at 3440m (day 4; 2 days) and 4371m (day 7; 2 days). These testing days were scheduled as part of a conservative acclimatization schedule [10] to mitigate acute mountain sickness and prevent the need for prophylactic acetazolamide, and were also necessary to complete a range of studies conducted during ascent. High-altitude medications (e.g., acetazolamide, dexamethasone) and oxygen were available at all times in case of an emergency. The majority of the research team then spent 3 weeks at the Pyramid Laboratory (5050m). Those conducting the study on Sherpa children left the group at Namche Bazaar and hiked first to Thame (3792m), and then to Khunde (3853m) to conduct testing over 2 weeks.

### Equipment logistics

Over six metric tons of equipment were transported to 5050m in a combination of Pelican cases (for delicate items; www.pelican.com), waterproof barrels, and duffel bags. Pelican cases were used to house fragile equipment throughout the trans-continental flights and erratic ground transport through the Himalaya. Over 50 porters (who were not tested) and 20 yaks were hired for the transport of our equipment. Approximately 30 K-size gas cylinders (~1500 kg in total) were transported to the Pyramid Laboratory 4–12 weeks in advance. The Sherpa and lowlander participants carried similar loads (i.e., just personal backpack) on ascent. Ultrasound equipment was carried in personal backpacks by the sonographers as their failure would have compromised nearly every study (Table 1).

### Equipment

Researchers will undoubtedly have their own equipment preferences based on economy and practicality. Some comment on our experiences with various equipment bears mention as the functionality and durability of equipment in the stresses of the field varies markedly.

#### Blood

Radiometer ABL90 FLEX (Radiometer, Copenhagen) and i-STAT (Abbot Point of Care, Princeton, New Jersey) devices were used for the measurement of arterial blood gases. The upright device (ABL90 FLEX) from Radiometer does function at altitude, but it constantly demanded recalibration and flushing due to blood clots in the line; a problem seldom encountered at sea-level. This device also required a constant temperature well above the ambient temperatures usually experienced at altitude. Fortunately, this was possible in certain areas of the lodge attached to the Pyramid Laboratory. The i-STAT device can be kept warm in a jacket pocket, uses a small amount of blood, and successfully processed ~300 samples in Kathmandu and upon ascent. However, at 5050m we did require ~25% more of the single-use disposable cartridges (model, EG6+) than anticipated due to clotting (which requires a new cartridge). Indeed, clotting was also an issue with venous blood collection and drug infusions. A large gauge needle/cannula is recommended as it can be difficult to maintain a patent cannula during infusions, and due to greater blood viscosity as well as reduced atmospheric pressure, filling vacutainers becomes quite problematic. As such, we recommend 18G needles and to double the size of vacutainers relative to that used at sea-level for a given volume of blood.

#### Ventilation

Some studies utilized a full PowerLab setup (ADInstruments, Colorado Springs) but the requirements for a constant, clean electrical supply are problematic. We used a Wright Analog Spirometer and a digital capnograph (EMMA capnograph, Masimo, Irvine, CA) to measure minute ventilation and end-tidal PCO_2_, respectively. These devices are robust, small, and require no AC power supply, and, together with SpO_2_, provide an index of ventilatory response to altitude [11]. It is important to realize the inaccuracy of pulse oximeters at SpO_2_ values below ~70%; hence values should be verified, if possible, with arterial blood sampling [12].

#### Ultrasound

Echocardiography was performed by two experienced sonographers using commercially available, portable ultrasound machines (Vivid Q, GE Healthcare, Piscataway, NJ, USA). These devices have a relatively short battery life (<45 minutes) so access to an AC power supply was required. Members of the research team have used these devices on numerous high-altitude expeditions, and have found them to be remarkably robust to the inhospitable environmental conditions. Peripheral (via brachial and superficial femoral arteries) and cerebral (via internal carotid and vertebral arteries) vascular assessments were completed with Duplex ultrasound (10Hz probe, 15L4, Terason t3200, Burlington, MA, USA), with vessel diameter and blood velocity measured offline at 30Hz. Data backups were performed daily to multiple portable encrypted solid state drives.

#### Microneurography

Studies of sympathetic nerve activity using microneurography are very rare at high-altitude; to our knowledge only three other studies have been completed [13–15]. In one of our studies multiunit microneurography recordings of sympathetic outflow in the peroneal nerve were successfully undertaken by two experienced microneurographers. We used a Nerve Traffic Analyser consisting of an electrode, a preamplifier and an electronic system (662C-3, Bioengineering of University of Iowa, Iowa City, IA). This system proved to be robust, surviving both the journey to and from the research station. Furthermore, despite the demanding environment for both researchers and participants, neural signal detection at high-altitude was good, with high signal to noise ratios, and very few problems caused by electrical interference. This was in contrast to problems encountered in Kathmandu that were associated with intermittent power supply and electrical interference.

### Protocols

Seventeen mechanistic study protocols, some with a number of sub-questions / hypotheses, were successfully completed at altitudes up to and including 5050m. These core studies focused on the mechanisms of cerebral blood flow regulation, iron metabolism, pulmonary pressures, intra-ocular pressures, cardiac function, neuromuscular fatigue, motor control, blood volume regulation, autonomic control and micro and macro vascular function. The studies are broadly defined here by those conducted during ascent and those conducted at 5050m (see Table 1 for further details).

#### Ascent studies

Using semi-mobile equipment and simple experimental designs six studies were completed during ascent to 5050m.

Temporary laboratories were situated in Kathmandu (1400m), Namche Bazaar (3440m) and Pheriche (4371m). The permanent laboratory was at 5050m. Barometric pressure, temperature, and humidity data were recorded daily at each laboratory and are summarized in Table 3.

**Table 3:** Environmental variables at each testing site.

Site	Altitude (m)	Temperature	Humidity
UBC	344	19 (3)	30(7)
Kathmandu	1400	22 (3)	42 (11)
Namche Bazaar	3440	14 (4)	39 (7)
Pheriche	4371	11 (3)	32(5)
Pyramid Laboratory	5050	9 (5)	29(6)

Mean (±SD). UBC = Kelowna, Pyramid Laboratory = Ev-K2-CNR Research Pyramid Laboratory.

For the presented data (see Results), radial artery blood gases were procured in the morning, in the fasted state, following at least 10-minutes supine rest at each location during the ascent. A 23-G self-filling catheter (SafePico, Radiometer) was advanced into the radial artery under local anesthesia (Lidocaine, 1.0%) and ultrasound guidance (Terason, uSmart 3300). Approximately 1mL of blood was withdrawn anaerobically and immediately assessed using an arterial blood gas analyzer (i-STAT) for PaO_2_, the partial pressure of arterial carbon dioxide (PaCO_2_), pH, and bicarbonate (HCO_3_^-^).

#### Studies at 5050m

**Fig 3:**
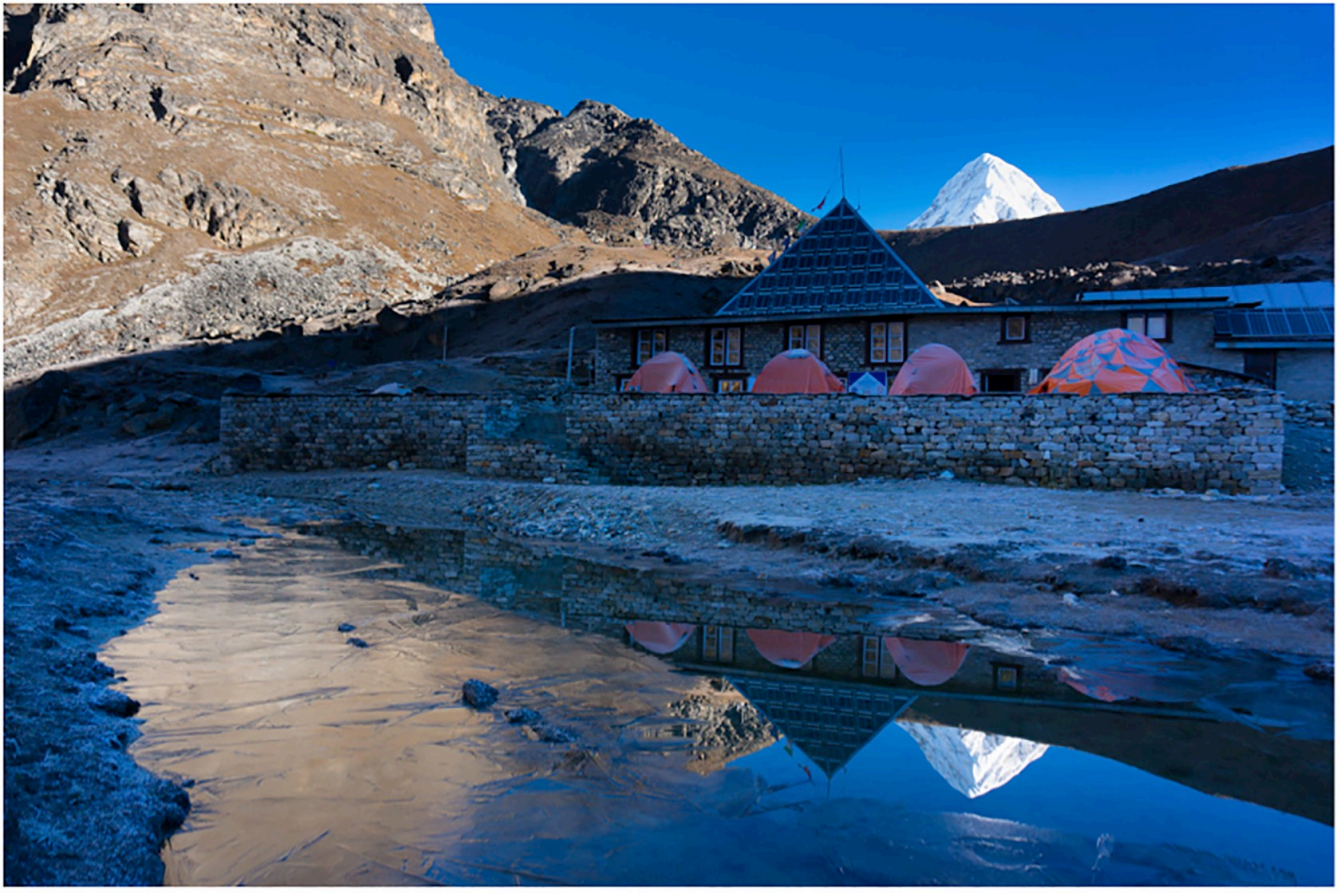
The Ev-K2-CNR laboratory, 5050m, Solokhumbu, Nepal.

The Ev-K2-CNR research laboratory at 5050m is one of the finest high-altitude research facilities in the world (Fig 3). An extensive battery bank charged by a solar array or diesel generator (during overcast weather) provided electricity routed through an uninterrupted power supply to maintain stable power as outages sometimes occurred. In total, this power enabled 2–5 fully functional laboratories to operate for 10–14 hours per day. Sophisticated blood gas analyzers (ABL90 Flex, Radiometer) were stored and operated in a temperature controlled room located in the main lodge. The adjoining lodge provided accommodation and food for up to 30 participants, with tents for additional persons.

#### Biological sample storage and transport

During both the ascent and laboratory studies at 5050m, blood samples were collected from an arm vein and quickly spun in a portable centrifuge (at 2000–3000 RPM, Drucker Diagnostics, Model 642VES, Port Matilda, PA). Serum and plasma were aliquoted into 2ml cryotubes and stored in liquid nitrogen (-196°C). Samples were brought from the field to Kathmandu and shipped to Canada on dry ice (-78.5°C; Marken Inc; temperature verified). Iron, transferrin saturation, and ferritin were measured by an accredited laboratory (Samyak Diagnostic, Kathmandu, Nepal; ISO 15189:2012).

#### Sample size estimates

Minimum subject sample sizes were determined *a priori* based on the specific study. Based on our previous exercise and high-altitude studies (e.g. [16–21]) adequate sample sizes for each of the outlined studies, accounting for potential subject dropout, were determined by related statistical power calculations whereby a power of 0.8 was assumed, and an alpha value of 0.05 was set (G*power). Depending on the variability of the primary outcome of each study (e.g., CBF, PASP, etc.), 8–30 participants were required.

#### Statistical analyses

The presented arterial blood gas data were analyzed with a linear mixed effects model utilizing a compound symmetry covariance matrix. The factors were Altitude and Race, with altitude as a repeated measure. Upon detection of a significant interaction (P<0.05), Bonferroni corrected post-hoc tests were utilized for pairwise comparisons. All statistical tests were performed with the Statistical Package for the Social Sciences (SPSS, V24).

## Results

**Fig 4:**
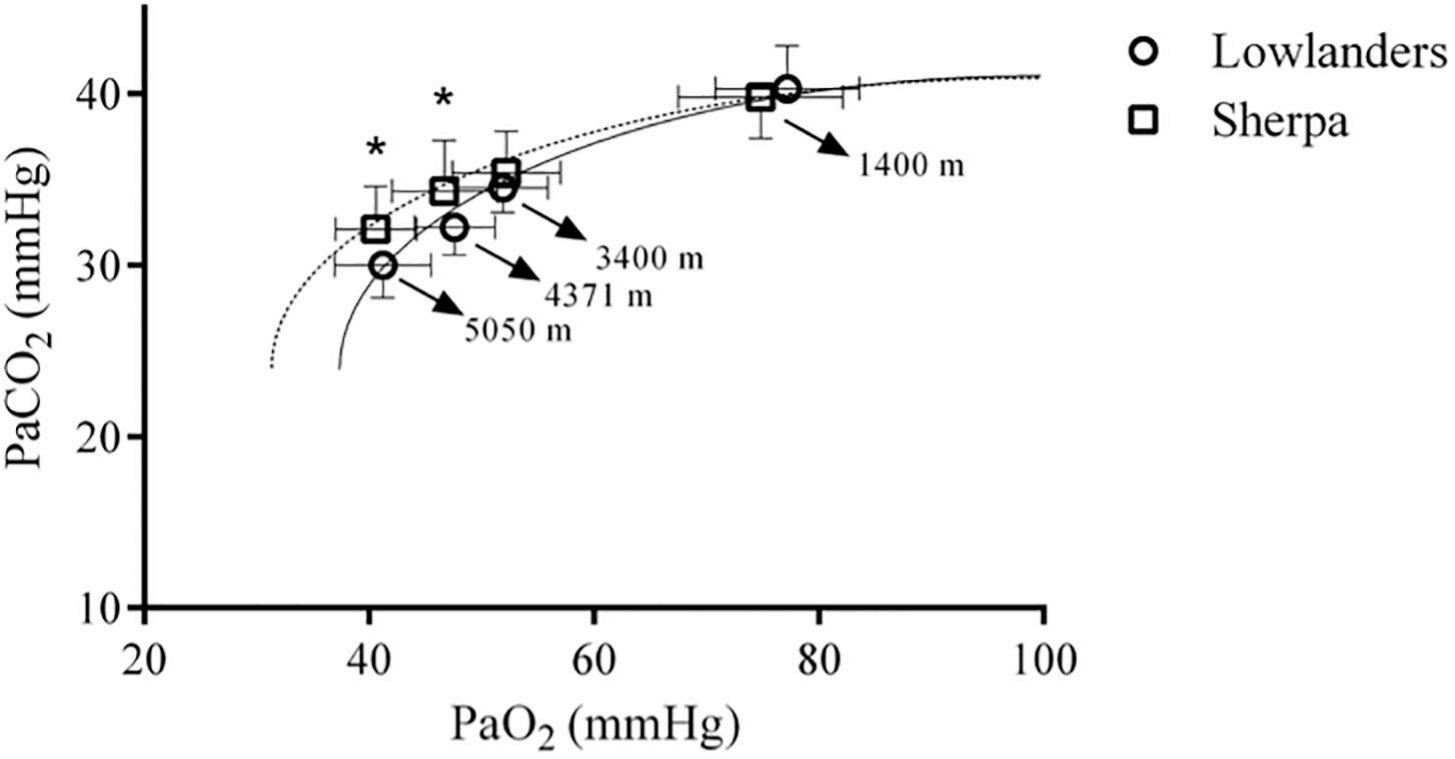
Rahn & Otis curves for Sherpa and lowlander upon ascent to altitude. Lowlanders are denoted by the open circle symbol (∘), and Sherpa by the open square symbol (□). Moving right to left, data are plotted from Kathmandu (1400m), Namche Bazaar (3400m), Pheriche (4371m), and the Pyramid Laboratory (5050m). * denotes a significant difference between Sherpa and lowlanders for PaCO_2_ at a given altitude (P<0.05).

Arterial blood gas data was successfully collected at each altitude on 21 lowlanders and 11 Sherpa. The results are summarized in Table 4. Changes in PaO_2_ and SaO_2_ were not different between Sherpa and lowlanders. At each altitude PaCO_2_ was decreased in both groups (Fig 4); however, PaCO_2_ was greater in Sherpa at Pheriche and the Pyramid Laboratory (P<0.05 for both). This, coupled with a lower [HCO_3_^-^] at Kathmandu and Namche Bazaar for Sherpa (P<0.05 for both), led to a main effect of race for pH (P<0.05). Indeed, the Sherpa were less alkalotic at each altitude than lowlanders.

**Table 4:** Arterial blood gas data throughout ascent.

		Kathmandu	Namche Bazaar	Pheriche	Pyramid Laboratory
PaO_2_ (mmHg)	*Altitude*, *P<0*.*001; Race*, *P = 0*.*489; Interaction*, *P = 0*.*649*				
	Lowlander	77.2±6.4	51.9±4.0*	47.6±3.6*	41.2±4.3*
	Sherpa	74.8±7.3	52.2±4.8*	46.7±4.7*	40.6±3.6*
SaO_2_ (%)	*Altitude*, *P<0*.*001; Race*, *P = 0*.*143; Interaction*, *P = 0*.*794*				
	Lowlander	95.4±1.2	87.4±2.6*	84.5±3.1*	78.9±4.8*
	Sherpa	94.5±2.0	86.7±3.3*	82.5±4.8*	77.4±4.2*
PaCO_2_ (mmHg)	*Altitude*, *P<0*.*001; Race*, *P = 0*.*043; Interaction*, *P = 0*.*008*				
	Lowlander	40.3±2.5	34.5±1.4*	32.2±1.6*†	30.0±1.9*†
	**Sherpa**	39.8±2.4	35.4±2.4*	34.3±3.0*	32.1±2.5*
HCO_3_^-^ (meq/L)	*Altitude*, *P<0*.*001; Race*, *P = 0*.*144; Interaction*, *P = 0*.*002*				
	Lowlander	26.3±1.4†	23.5±1.3*†	21.5±1.4*	21.4±1.5*
	Sherpa	24.6±1.2	22.3±1.7*	21.5±1.9*	21.7±2.1*
pH	*Altitude*, *P<0*.*001; Race*, *P<0*.*001; Interaction*, *P = 0*.*956*				
	**Lowlander**	7.42±0.02	7.44±0.02*	7.43±0.02	7.46±0.02*
	Sherpa	7.40±0.02	7.41±0.02*	7.40±0.02	7.44±0.02*

**Bolded** “Lowlander” or “Sherpa” denotes greater values across altitudes, P<0.05 (main effect);

* denotes a difference from Kathmandu, P<0.05;

^†^ denotes a difference between Sherpa and Lowlanders, P<0.05 (Pairwise comparison).

Pyramid Laboratory = Ev-K2-CNR Research Laboratory.

Note that no lowlander or Sherpa children were included in the ascent studies.

The data presented are based on adults only.

## Discussion

Although there were numerous setbacks over the course of the expedition that ranged from the typical illnesses experienced in developing countries to tanks of pure nitrogen instead of 15% oxygen arriving at 5050m, every planned study was successfully completed. Nevertheless, number of potentially confounding factors still bear consideration to help inform future expeditions to high-altitude.

### Sherpa studies and retention of altitude acclimatization

A consistent observation is that following descent to lower elevations, humans retain some of the acclimatization to high-altitude for some time and show a much faster acclimatization upon re-ascent [22,23]. For example, in lowlanders, it was recently reported that, after descending to low altitude from 5260 m for one or three weeks, physiological evidence of acclimatization persisted upon returning to 5260 m. These changes were manifested by lower AMS incidence, retention of exercise performance, and, to some extent, ventilatory acclimatization (higher PaO_2_ and lowered PaCO_2_) and cognitive performance [23]. The questions arises: Would the Sherpa adults who descended to lower elevations for 5–15 days display some form of altitude retention and hence be a methodological flaw in our experimental design? There are three important points to this question. First, our fundamental design was to compare Sherpa to lowlanders during ascent to altitude. With acknowledgment that altitude retention is possible in our Sherpa group, we felt this was an important comparison to make to better understand how lowlanders respond to altitude when compared to the Sherpa. Second, people living at high-altitude regularly ascend and descend in the mountainous environment and hence our design is of practical relevance. Finally, in many of the studies in this investigation, by including an additional group of Sherpa who did not descend, we have a further comparison of those without the potential influence of descent. This final point is also important in that it allows for an improved ability to dissociate acclimatization from evolutionary differences that may exist between the lowlander and Sherpa participants. In other words, if a difference is not observed between lowlanders and the ascending Sherpa, but is observed between lowlanders and the at-altitude Sherpa, the difference has likely manifested as a result of acclimatization. If a difference exists between lowlanders and both Sherpa groups, this is more likely to have manifested as a result of evolutionary adaptation.

### Laboratory vs. field

For obvious reasons of ecological validity, we elected to undergo a comparative field study between lowlanders and Sherpa during ascent and over time at 5050m. Although it would not have been possible to complete such a study with our sample size in a hypobaric chamber, there are many uncontrolled variables during a field study. These factors include variable temperature, sample size, diet, exercise / physical inactivity, etc. Of note, in the present set of studies, these factors were common across participants during ascent and at 5050m. In some ways, it is almost impossible to fully control the intensity and duration of ascent on an individual level; however, in other ways, this approach is more realistic of a typical trek and high altitude where individuals typically walk in groups. Nevertheless, all participants were encouraged to trek at a conservative pace to avoid overexertion and limit the risk of altitude illness.

### Multiple publications

This research study encompassed many collaborators and a range of observational, invasive and mechanistic physiological experiments with *a priori* determined aims and hypotheses. As such, the results will be partitioned into discrete papers led by the coordinating principle investigator of the experiments. The coordination of large-scale high-altitude research requires planning, organization and management that typically begins 2–5 years in advance. Expeditions are expensive and require substantial funding. They demand a coordinated approach by a compatible research team, and the logistical and financial challenges of personnel and equipment transport must be tackled. The transportation of expensive and fragile equipment to high-altitude regions is a significant final hurdle because conventional carriage is impossible and there may exist difficulties with importation tax and customs. It is for these reasons that high-altitude field studies have normally included multiple experimental questions and their corollary publications, often yielding >10 papers from a single expedition (recent examples include: AltitudeOMICS [23]; Caudwell Xtreme Everest [10]). Such a research design is sometimes criticized for duplication or overlap of data, but is defensible providing that that any duplication of data is acknowledged and that publications are not intentionally partitioned, but rather best packaged to address their respective *a priori* hypotheses. Moreover, because of unknown complications and related risks of conducting research at altitude, many of the planned experiments are unsuccessful; hence, they are never published. Nonetheless, duplication of data will be limited to variables germane to multiple studies, for example, data from arterial and venous blood sampling, pulmonary function testing, and standard cardiovascular variables such as blood pressure and heart rate.

### Translational opportunities

Hypoxemia is commonplace amongst critically and chronically ill patients with optimal management strategies remaining unclear. The approach of investigating mountaineers exposed to hypoxia at high-altitude offers the advantage that a relatively homogeneous and healthy population can be studied, in contrast to the heterogeneous and generally less healthy patient population typically observed in critical care units. While some of the linkages between altitude-related studies and critical pathologies remain unclear, studies have shed new light on our understanding of the pathogenesis of various hypoxia-related diseases [e.g., pulmonary edema, acute respiratory distress syndrome, etc; see [2] for review]. The important clinical links between oxygen, iron availability and pulmonary pressure regulation have been recently documented [24,25]. By progressive investigations in the Sherpa, new insight into the mechanisms that lead to beneficial adaptation may further develop into individualized treatment strategies. Continuing fieldwork in high-altitude residents is urgent since modernization and migration are changing the traditional ways of life and patterns of exposure to the environment among highlanders everywhere. The study of humans at altitude will moreover facilitate explication of the processes by which humans have evolved to their environments.

A further translatable opportunity that such an expedition offers is career development of team members. This education occurs on many levels, ranging from undergraduate to graduate students who, because of the unique training environment, may collect data for presentations and publications. Perhaps more importantly, the interdisciplinary/collaborative aspect of this kind of field research provides invaluable experience when things seldom go as planned (i.e., it fosters ingenuity, adaptability, team work, etc.). Moreover, there were intentionally many early career researchers, who led their own independent projects within the supported group. As such, their careers will be further enhanced by the success and networking proffered by this expedition. The majority of the team are involved with basic science or medical education; thereafter, both the experiences and the data collected during these trips will be used in a range of formal and informal teaching settings.

### Sherpa vs. lowlander arterial blood gases during ascent to altitude

A recent review [6] concluded, following the assessment of 21 related papers, that the hypoxic ventilatory response of Tibetans/Sherpa was not different from lowlanders. Early studies had suggested a blunting of the hypoxic ventilatory response in Sherpa [26,27], whereas other reports indicate they may ventilate more at rest [28]. Inevitably, methodological differences between studies, the acclimatization process, and differences in altitude at which Sherpa and lowlanders were assessed have all been considered contributory factors to these inconsistent findings. Further, measurements of minute ventilation (e.g., spirometry) may not be purely representative of differences in alveolar ventilation between groups, especially considering the noted differences in lung volumes [29] and diffusion capacity [30]. However, PaCO_2_ provides an effective means to index alveolar ventilation that accounts for potential differences in volumes and diffusion capacities [31–36].

$${PaCO}_{2}\;=\;\frac{{VCO}_{2}}{V_{A}}$$

Where V_A_ represents alveolar ventilation and VCO_2_ is metabolic CO_2_ production. Thus, Rahn & Otis curves were plotted for the purposes of comparing effective alveolar ventilation between the Sherpa and lowlander groups across each altitude (Fig 4). As depicted in the figure, it can be seen that below a PaO_2_ of ~50mmHg, lowlanders have a downward shift in the curve. This would indicate that alveolar ventilation is greater in lowlanders under the assumption that VCO_2_ is not different. Given the lower VO_2_ of Sherpa [6,37], and therefore, VCO_2_, differences in alveolar ventilation are underrepresented by the plotted lines. Overall, our arterial blood gas data, in line with previous small sample size studies [27], indicate that subsequent to a short de-acclimatization period, Sherpa possess a lower alveolar ventilation (and are hence less alkalotic) than lowlanders during the same ascent profile. The lower alveolar ventilation cannot be attributed to central chemoreceptor drive to breathe as arterial pH was lower, which should lead to a greater ventilation (under the assumption of comparable changes in brain tissue pH at the level of the brainstem). Therefore, it is more likely that some form of peripheral chemoreceptor-mediated adaptation, may be responsible for this difference; however, we acknowledge the regulation of breathing at high-altitude is highly complex [38] and could be altered at the level of afferent input [39] central integration [40], and/or efferent output [41].

### Lessons learned

Invasive human physiology research necessitates a large team of compatible, expert individuals to complete intricate experimental paradigms. This is especially true of field studies where it seems that if something can go wrong, it generally will. Our 2016 expedition overcame numerous problems that could have easily thwarted what was ultimately a success. It is worth describing herein some of the factors that contributed to each of our planned studies being completed, if only narrowly, to help with the successful planning of future expeditions.

#### Local support

There were few moments when the expedition’s Sirdar, Nima Sherpa of Khunde, was not on a cellular phone coordinating logistics ranging from helicopters to delivery of several tons of compressed gas canisters by human porters to recruitment and scheduling of local study volunteers. Particularly given the profound differences in language, cultural norms and business practice, it is imperative to have an efficient and reliable local manager.

#### Electricity

It is obvious that electricity is necessary, but the importance of a stable, clean power source needed to run sophisticated equipment cannot be overstated. For example, despite our electrician designing two uninterrupted power supplies, we caused a number of power outages, in addition to two contained electrical fires while running baseline studies in a Kathmandu hotel. Thus, a competent tradesperson or engineer is integral to the success of such an expedition.

#### Health

Despite the improbability of non-hypoxia related emergency illness (appendicitis, for example), evacuation plans and appropriate clinical counterparts are required to aptly handle such situations. In general, the effect of illness on team morale is severe. It is thus imperative to provide good medical care, but also quality food and treats, which together greatly contribute to the spirit and motivation of the team.

#### Safety

For our previous expeditions in 2008 and 2012, all compressed gases were shipped from either Australia or Canada to Nepal, which, while costly and time consuming, guaranteed the correct gas content. Compressed gases were procured from India in this 2016 expedition and shipped ahead of the research team to the Pyramid Laboratory. These tanks were found to vary markedly from their stated composition—more than one was pure nitrogen, which could have been calamitous had this gas been accidently given to a volunteer. Because of likely impairment in cognitive function at altitude, it is highly recommended that more than one investigator confirm the exact mix of compressed gases and double-check correct dosing for any pharmacological intervention.

## Conclusion

The 2016 UBC Expedition was comprised of seventeen studies on five distinct cohorts: 1) 30 lowlander adults; 2) 12 Sherpa adults who de-acclimatized at 1400m for 5–15 days; 3) 12 Sherpa adults who had not recently descended below 3500m; 4) 57 Sherpa children; 5) 30 age and BMI-matched lowlander children tested in Canada at 344m. Studies were conducted both during a nine-day trekking ascent to 5050m and during three weeks at 5050m, which focused on cardiovascular, cerebrovascular, cardiopulmonary and neuromuscular aspects of human physiological responses to acclimatization. The findings from this study will be reported in approximately seventeen ensuing publications according to their respective *a priori* hypotheses.
